# Two decades of Iranian midwives' activities as a health care provider under supervision in a multidisciplinary team in reducing maternal mortality

**DOI:** 10.1186/s12978-021-01100-3

**Published:** 2021-02-12

**Authors:** Mojgan Mirghafourvand, Shahla Khosravi, Jafar Sadegh Tabrizi, Azam Mohammadi, Parvin Abedi

**Affiliations:** 1 Department of Midwifery, Social Determinants of Health Research Center, Tabriz University of Medical Sciences, Tabriz, Iran; 2 Department of Community Medicine, Faculty Member of Medicine School, Tehran University of Medical Sciences, Tehran, Iran; 3 Health Management Research Institute, Tabriz University of Medical Sciences, Tabriz, Iran; 4 Tehran University of Medical Sciences, Tehran, Iran; 5 Menopause Andropause Research Center, Midwifery Department, Ahvaz Jundishapur University of Medical Sciences, Ahvaz, Iran

**Keywords:** Iranian midwives, Maternal mortality, Health care provider

## Abstract

Iran is amongst the countries that have achieved the fifth goal of the United Nations Millennium Development Goal. The maternal mortality ratio (MMR) in Iran has declined from 48 cases per 100,000 in 2000 to 16 cases per 100,000 in 2017, showing an annual decline rate of about 6.3%. In the International Year of the Nurse and the Midwife (year 2020), this commentary highlights two decades of Iranian midwives' activities as a health care provider under supervision in a multidisciplinary team in reducing maternal mortality.

Human development indicates achieving full health, knowledge, and ability to handle and manage life; and it is mainly intended to help and profit human beings [1]. One of the principal points in human development is to consider maternal health because around 808 women die every single day as a result of pregnancy and its complications [2]. Woman in poor or low-income countries are facing the risk of dying during pregnancy 130 times more than women in developed countries [2, 3].

A fundamental measure taken to react against the imposing challenges of international development was the Millennium Development Goals (MDGs), committed to helping achieve the eight basic goals by 2015 [4]. Given the importance of maternal mortality, the fifth goal of the UN's MDG (adopted by 189 countries worldwide) explicitly addressed this issue and tried to reduce maternal mortality up to 75% during 1990 and 2015 [5]. As with this goal, the maternal mortality ratio (MMR) over the course of 25 years had to be reduced by at least 5.5% per year to achieve the ultimate goal [6].

According to the analysis of data available at the Global Burden of Disease (GBD) website, the maternal mortality rate in Iran has declined from 40 deaths per 100,000 live births in 1990 to 14 in 2015, indicating a 1.36% decrease on average. This decline has been constant in Iran so that the trend of mortality (without a peak period) has been steadily declining, with the raw number of maternal deaths from 123 in 1990 to 25 deaths in 2015. Such figures indicate on average a decline of about 3.7 units annually [7], meaning that Iran is amongst the countries that have achieved the fifth goal of the UN's MDG [5]. As reported by the WHO, the maternal mortality ratio (MMR) has declined from 48 cases per 100,000 in 2000 to 16 cases per 100,000 in 2017, showing an annual decline rate of about 6.3% [8].

The quality and quantity of health and therapeutic cares for mothers contribute significantly to prevent maternal mortality rate [9], while yet the performance of skilled and educated health care providers is imperative. Taking comprehensive coverage of maternal care during pregnancy and childbirth by skilled and trained personnel in health and therapeutic centers is significantly associated with a reduced rate of maternal mortality [10]. Midwives have the skills required to extend the strengths of individuals and communities to improve therapeutic outcomes. As part of multidisciplinary team, they can provide valuable assistance to reduce risk factors. They contribute to the prevention, early screening/diagnosis, and treatment as well as in health promotion [11]. The results of numerous international studies show that the continued health care by midwives during pregnancy and at childbirth improves both maternal and neonatal health and raises the level of maternal satisfaction [12]. Of course, one of the reasons directly affect the decline in maternal mortality in Iran is decreased fertility rate due to family planning programs [13]. Family planning contributes to decreasing maternal mortality through reducing the number of births and, therefore, the number of times a woman is exposed to the risk of mortality [14].

The statistics reveal a notable increase in the abundance of human resources for health, including midwives in Iran [15]. In Iran, midwives are the largest group of health care providers in health centers [11], and about 33,208 midwives are operating in the Iranian health system [15] at several levels of management (Ministry of Health and Medical Education at the policymaking and management level), education (training midwifery undergraduates, masters, and doctorates, and health workers) and as a member of health care team and under supervision of obstetricians or General Practitioner in "headquarters", "maternal programs", "healthy reproduction and population program", "breastfeeding plan", "urban and rural health centers" and "birth facilities".

At health centers, 10,517 midwives as community health workers offer specialized services in the form of reproductive health programs with use of National Guidelines provided for them and electronic health registry under medical universities supervision in collaboration with other members of the health care system, including gynecologists, general practitioners and health professionals. The foremost activities of the reproductive health program include "monitoring the implementation of integrated maternal health care to increase the quantitative and qualitative performance of care measures during pregnancy. Midwives as one of the members of multidisciplinary team in the maternal mortality committees in Iran usually help in data gathering and narrating of the maternal mortality reports with the help of obstetricians and sometimes introducing the cases. Midwives also provide educational, counseling, and care services before, during, and after pregnancy for all women.

About 20,000 midwives work in medical centers and offer valuable services with normal delivery and midwifery emergency services. Also, there are currently 206 and 86 active birth facilities respectively in cities and rural areas. In addition, a significant number of midwives work in private clinics and offer reproductive health services including preventive, counseling, and treatment services in their clinics. Since the World Health Organization (WHO) has reported that there is a need for 18.1 midwives per 100,000 people [16], it appears that Iran is encountering a deficiency in the number of employed midwives, marking the necessity to attract unemployed midwives in Iran.

With the WHO declaring that investing in midwifery can save the lives of millions of women and infants [17] and since the WHO has declared 2020 as the International Year of the Nurse and the Midwife, investing in the midwifery sector will significantly contribute to the global health care at a fast, cost-effective and high-quality manner [16]. Investment in this sector and providing required services by authorities is, therefore, essential. It is, thereby, recommended that national authorities take necessary measures to reduce the rate of maternal mortality and promote the health of mothers and infants, and eventually society by magnifying the role of midwives and allocating more funds to employ trained midwifery forces and make the best use of these inexpensive empowered forces.

## Data Availability

Not applicable.

## Abbreviations

MMR: Maternal mortality ratio
MDG: Millennium Development Goals
UN: United Nations
GBD: Global Burden of Disease
WHO: World Health Organization
